# The role of *MLH1*, *MSH2* and *MSH6* in the development of multiple colorectal cancers

**DOI:** 10.1038/sj.bjc.6602708

**Published:** 2005-08-02

**Authors:** D A Lawes, T Pearson, S SenGupta, P B Boulos

**Affiliations:** 1 Department of Surgery, Royal Free and University College London, 2nd Floor 67-73 Riding House Street, London, W1W 7EJ, UK

**Keywords:** microsatellite instability, synchronous colorectal cancer, metachronous colorectal cancer

## Abstract

There is increased incidence of microsatellite instability (MSI) in patients who develop multiple primary colorectal cancers (CRC), although the association with hereditary nonpolyposis colon cancer (HNPCC) is unclear. This study aims to evaluate the underlying genetic cause of MSI in these patients. Microsatellite instability was investigated in 111 paraffin-embedded CRCs obtained from 78 patients with metachronous and synchronous cancers, and a control group consisting of 74 cancers from patients with a single CRC. Tumours were classified as high level (MSI-H), low level (MSI-L) or stable (MSS). *MLH1*, *MSH2* and *MSH6* gene expression was measured by immunohistochemistry. Methylation of the *MLH1* promoter region was evaluated in MSI-H cancers that failed to express *MLH1*, and mutational analysis performed in MSI-H samples that expressed *MLH1*, *MSH2* and *MSH6* proteins. The frequency of MSI-H was significantly greater in the multiple, 58 out of 111 (52%), compared to the single cancers, 10 out of 74 (13.5%), *P*<0.01. Of the 32 patients from whom two or more cancers were analysed, eight (25%) demonstrated MSI-H in both cancers, 13 (41%) demonstrated MSI-H in one cancer and 11 (34%) failed to demonstrate any MSI-H. MSI-H single cancers failed to express *MLH1* or *MSH2* in seven out of nine (78%) cases and MSI-L/MSS cancers failed to express *MLH1* or *MSH2* in one out of 45 (2.2%) cases, all cancers expressed *MSH6*. MSI-H multiple cancers failed to express *MLH1* or *MSH2* in 21 out of 43 (48%) cases and MSI-L/MSS cancers failed to express *MLH1* or *MSH2* in four out of 32 (12.5%) cases. *MSH6* expression was lost in five MSI-H multiple cancers, four of which also failed to express *MLH1* or *MSH2*. Loss of expression of the same mismatch repair (MMR) gene was identified in both cancers from six out of 19 (31%) patients. Methylation was identified in 11 out of 17 (65%) multiple and three out of six (50%) single MSI-H cancers that failed to express *MLH1*. Mutational analysis of 10 MSI-H multiple cancers that expressed *MLH1*, *MSH2* and *MSH6* failed to demonstrate mutations in the *MLH1* or *MSH2* genes. We suggest that, although MSI-H is more commonly identified in those with multiple colorectal cancers, this does not commonly arise from a classical HNPCC pathway.

Colorectal cancer (CRC) develops via two distinct genetic pathways. The ‘suppressor’ pathway involves loss of function of the tumour suppressor genes APC, DCC and p53, and the activation of the proto-oncogene k-ras. This accounts for 85% of sporadic CRC (Ilyas *et al*, 1999) and for cancers associated with familial adenomatous polyposis (FAP), that constitute 1% of all CRC (Vasen, 2000). In the ‘mutator’ pathway, mutations in mismatch repair (MMR) genes such as *MLH1*, *MSH2*, *MSH6*, PMS1 and PMS2 lead to microsatellite instability (MSI) in genes such as TGF*β*RII, ILGF, E2F-4 and BAX, whose coding regions are closely associated with microsatellite DNA (Markowitz *et al*, 1995; Souza *et al*, 1996, 1997; Rampino *et al*, 1997). Microsatellite instability is detected in 15% of sporadic CRC and 90% of cancers from patients with hereditary nonpolyposis colon cancer (HNPCC), which accounts for 1–2% of all CRC (Aaltonen *et al*, 1998; Percesepe *et al*, 2001; Samowitz *et al*, 2001).

Microsatellite instability is classified according to the level of instability detected as high (MSI-H), low (MSI-L) or stable (MSS) (Boland *et al*, 1998). In MSI-H sporadic cancers, there is predominantly loss of *MLH1* function (Thibodeau *et al*, 1998), caused by transcriptional silencing of the gene brought about by methylation of the *MLH1* promoter region (Herman *et al*, 1998). Hereditary nonpolyposis colon cancer associated cancers arise from *MLH1* and *MSH2* germline mutations and rarely involve mutations of other genes (Liu *et al*, 1996; Peltomaki and Vasen, 1997) or *MLH1* promoter region methylation (Wheeler *et al*, 2000; Potocnik *et al*, 2001; Yamamoto *et al*, 2002).

At least half the patients with HNPCC will develop a metchronous CRC when treated by a standard colorectal resection (Lynch, 1996), and the Bethesda criteria, introduced to identify those with HNPCC advise MSI analysis in patients with multiple CRC (Rodriguez-Bigas *et al*, 1997). Microsatellite instability has been reported in 33–89% of cancers from patients with multiple cancers (Horii *et al*, 1994; Sengupta *et al*, 1997), including studies that excluded HNPCC clinically by the Amsterdam criteria (Brown *et al*, 1998; Masubuchi *et al*, 1999; Yamashita *et al*, 2000), although these results have been disputed (Pedroni *et al*, 1999). The Amsterdam criteria were not sufficiently selective (Beck *et al*, 1997), since the more rigorous Amsterdam criteria II (Vasen *et al*, 1999) and atypical HNPCC phenotypes associated with mutations in other MMR genes such as *MSH6* (Wagner *et al*, 2001) were not considered. The underlying genetic cause in patients who develop metachronous or synchronous cancers is not clear, germline mutations in *MLH1* and *MSH2* account for 90% of cases of HNPCC, with mutations in *MSH6* and PMS2 accounting for the remainder (Giardiello *et al*, 2001).

This study investigates the incidence of MSI in patients from the general population with multiple primary colorectal cancers, and the relevance of *MLH1*, *MSH2* and *MSH6* gene expression in identifying those patients who may have HNPCC.

## MATERIALS AND METHODS

### Patients

From the Thames cancer registry, 754 patients who developed either synchronous (393) or metachronous (361) colorectal cancers between 1972 and 1997 were identified, 387 females and 367 males, with a median age at diagnosis of 70 (range 32–94) years. There were 986 cancers in the left colon and 694 cancers in the right colon. The pathology departments of all hospitals identified by the registry were contacted, 12 of these agreed to supply tissue for this study and provided us with 111 samples of paraffin-embedded cancer tissue and corresponding normal mucosa from 78 patients (44 females, 34 males, median age 72 (range 35–91) years). A cancer was synchronous when detected within 2 years of the first (index) cancer, and metachronous when it occurred later. There were 52 synchronous cancers from 29 patients and 59 metachronous cancers from 49 patients, with two or more cancers from 32 patients available for analysis. There were 56 cancers in the left colon and 51 in the right side of the colon; the site of origin of four cancers was undetermined. A control group of 74 patients (31 females, 43 males, median age 74, range 38–100 years) with single CRC resected at least 7 years earlier was identified. There were 42 cancers in the left colon and 29 in the right colon; the site of origin of three cancers was undetermined. Paraffin-embedded cancer tissue and the corresponding normal mucosa were obtained from the resected specimens. Regional ethics committee approval for this study was obtained on the condition that all data were anonymous and that contact with patients to obtain family history was strictly prohibited.

### Analysis of MSI

DNA was extracted from the paraffin-embedded tissue using a standard technique (Sengupta *et al*, 1997) and microsatellite DNA was amplified by polymerase chain reaction (PCR) at the mononucleotide loci Bat 26, Bat 25 and Bat 40, and the dinucleotide loci D2S123, D5S346 and D17S250. Each PCR reaction mixture consisted of: 2′-deoxynucleoside 5′-triphosphates 0.2 mmol l^−1^ (Pharmacia, St Albans, UK); 1 × PCR buffer containing Tris-HCl 50 mmol l^−1^ (pH 9); KCL 50 mmol l^−1^; MgCl_2_ 7 mmol l^−1^; (NH_4_)_2_SO_4_ 16 mmol l^−1^ (HT Biotechnologies, Cambridge, UK): 0.5 U SuperTaq poymerase (HT Biotechnologies); 25 pmol of each primer and 100 ng of genomic DNA and sterile water to make a uniform volume of 50 *μ*l. The reaction was performed using an Omnigene thermal cycler (Hybaid, Teddington, UK). PCR products were analysed by single-stranded conformational polymorphism (SSCP) on a multiphor II system (Amersham Pharmacia Biotech) using an ExcelGel® DNA Analysis Kit (Amersham Pharmacia Biotech) and a Plus One ™ DNA silver staining kit (Amersham Pharmacia Biotech) on a Hoefer automated gel stainer. MSI was identified by the presence of a characteristic ladder pattern in tumour DNA but not in the corresponding normal mucosa. Cancers which demonstrated MSI in >30% of the loci investigated were designated as MSI-H, those with MSI in <30% of the loci were considered MSI-L and those failing to demonstrate instability at any locus were classified as MSS, in accordance with the guidelines laid down by the National Cancer Institute workshop on MSI (Boland *et al*, 1998). Cancers were categorised as MSI-H, while MSI-L and MSS were grouped together.

### Immunohistochemistry (IHC)

Formalin-fixed, paraffin-embedded cancer tissue sections 5 *μ*m thick were mounted on superfrost slides (BDH Laboratory Supplies) and held at 37°C overnight. Paraffin was removed and the tissue rehydrated using xylene and ethanol. Slides were subjected to microwave antigen retrieval in 10 mM citrate buffer (pH 6) at 85°C for 35 min and cooled in phosphate-buffered saline (PBS), pH 7.4 (Sigma). Endogenous peroxidase activity was blocked with 2% hydrogen peroxide in methanol and slides were washed with PBS prior to overnight incubation with the appropriate antibody at a dilution of 1 : 100. Commercially available monoclonal antibodies against the nuclear proteins *MLH1* (PharMingen International, Clone G168-15), *MSH2* (Calbiochem-Novabiochem International, Clone FE11) and *MSH6* (Serotec, Clone GTBP.P1/2.D4) were applied, followed by staining with Strept ABC complex/HRP Duet kit (DAKO Ltd, Denmark) in conjunction with diamino benzedene (DAB) 180 mg in 300 ml PBS with 300 *μ*l H_2_O_2_. Tissues were counterstained with Mayer's haematoxylin prior to mounting with DePex (BDH, Poole, UK). Slide scoring was performed without prior knowledge of MSI status. Loss of expression was determined by failure of the cancer cell nuclei, but not the normal mucosa or stromal cells to stain. Sections that did not stain or showed equivocal staining were excluded from further analysis.

### Methylation analysis

Methylation-specific PCR (MSP) following bisulfite modification was performed on samples that failed to express the *MLH1* gene using published primer sequences (Herman *et al*, 1998) designed to amplify either the methylated or unmethylated *MLH1* promoter region. For bisulphite modification, 5 *μ*l genomic DNA (100–350 ng of genomic DNA) was incubated for 10 min at 37°C with 1 *μ*l of salmon sperm DNA and 1 *μ*l of 10 N NaOH, and made up to 50 *μ*l with deionised H_2_O. Freshly prepared 10 mM hydroquinone (30 *μ*l) (Sigma) and 3 M NaHSO_3_^−^ (520 *μ*l) (Sigma) were added, covered with a layer of mineral oil and incubated at 50°C for 18 h. DNA was cleaned using the QIAquick Gel Extraction Kit (Qiagen) in accordance with the manufacturer's instructions. The clean DNA was incubated with 1.5 *μ*l 10 M NaOH at room temperature for 10 min (final concentration 0.3 M) prior to incubation at −30°C overnight with 1 *μ*l of glycogen (Ambion), 5 *μ*l of 3 M sodium acetate and 125 *μ*l of 100% ethanol. DNA was subsequently washed with 70% ethanol, air dried and re-suspended in sterile, deionised H_2_O. Following MSP, the PCR products were analysed using 2% agarose in Tris-borate-EDTA gel containing ethidium bromide at a concentration of 0.05 mg per 100 ml (BDH-Merck) and visualised under ultraviolet light.

### Mutational analysis

Mutational analysis was performed on multiple cancer samples with MSI-H that expressed the *MLH1* and *MSH2* proteins, as demonstrated by immunohistochemistry. All 19 *MLH1* and 16 *MSH2* exons underwent PCR amplification using published primer sequences (Kolodner *et al*, 1994; Wijnen *et al*, 1996) and were analysed by SSCP and silver staining.

### Statistical analysis

The age at diagnosis of the cancers in the groups was compared by Mann–Whitney *U*-test, while categorical data were compared by Fisher's exact test. All statistical analysis was performed using GraphPad InStat version 3.00 for Windows 95, GraphPad Software, San Diego, CA USA, www.graphpad.com.

## RESULTS

The study group was representative of the Thames cancer registry data. The age at diagnosis, (*P*=0.11), gender (*P*=0.4) and site distribution (*P*=0.22) of the study group were comparable to those of the whole group in the Registry. There were proportionally more patients with metachronous CRCs in the study group, compared to the Registry (*P*=0.012) (Table 1).

### Microsatellite status

MSI-H was detected in 10 (13.5%). single cancers, MSI-L in 19 (25.5%) and MSS in 45 (61%) cancers. There was no difference between the ages of patients with MSI-H (median 80, range 68–89 years) and with MSI-L/MSS (73.5, range 31–100 years) (*P*=0.2). There were more female patients with MSI-H (8 *vs* 2) than with MSI-L/MSS (23 *vs* 41) (*P*=0.01). MSI-H was detected in 58 (52%) multiple cancers, a significantly higher incidence than in the single cancers (*P*<0.001), MSI-L in 29 (26%) and MSS in 24 (22%) multiple cancers. The ages of patients with MSI-H multiple cancers (72.5, range 40–87 years) were similar to those in the MSI-L/MSS group (median 73, range 35–91 years), *P*=0.78. There was no difference in the sex distribution in MSI-H (30 females *vs* 28 males) and in MSI-L/MSS (30 females *vs* 23 males) cancers (*P*=0.7).

In 46 patients with multiple cancers, only a single cancer was available for analysis, 29 (63%) demonstrated MSI-H (median 71, range 53–87 years) and 17 (37%) MSI-L/MSS (median 73 years, range 53–87); the ages were not different in these groups (*P*=0.9). There was no difference in MSI-H in metachronous (35/62) or synchronous (23/49) cancers (*P*=0.3). In 32 patients, the MSI status of two or more cancers obtained from a single patient was available; in eight, MSI-H was detected in both cancers (one of whom demonstrated MSI-H in three synchronous cancers); in 13 MSI-H was identified in a single cancer from a pair and MSI-L/MSS in the corresponding cancer, and in 11 patients both cancers demonstrated MSI-L/MSS. There was no difference between the groups in the ages at diagnosis (Table 2).

### Immunohistochemistry

Of the single cancers, 19 paraffin-embedded tissue samples were inadequate for immunohistochemistry. Of nine MSI-H cancers, six failed to express *MLH1*, one failed to express *MSH2*, and two expressed *MLH1* and *MSH2*. One of the MSI-L/MSS cancers failed to express *MSH2* and the remaining 45 expressed both *MLH1* and *MSH2*. All 55 cancers expressed *MSH6* (Table 3). Of the multiple cancers, 36 paraffin-embedded samples were inadequate for immunohistochemistry. Of 43 MSI-H cancers, 14 failed to express the *MLH1*, seven failed to express *MSH2*, while 22 expressed *MLH1* and *MSH2*. Of 32 MSI-L/MSS cancers, 28 expressed *MLH1* and *MSH2*, three failed to express *MLH1* and one failed to express *MSH2*. Five MSI-H cancers failed to express the *MSH6*, of which one failed to express *MLH1*, three failed to express *MSH2* and only one cancer failed to express *MSH6* alone (Table 3).

There were 19 paired multiple cancers available for immunohistochemistry. In seven patients, both cancers demonstrated MSI-H; three failed to express *MSH2* and two failed to express *MLH1* in either cancer. One patient with three MSI-H synchronous cancers showed normal *MLH1*, *MSH2* and *MSH6* expression in their cancers, the last patient demonstrated loss of *MLH1* in one tumour, but *MLH1*, *MSH2* and *MSH6* expression in the other. *MSH6* expression was lost in two cancers that also failed to express *MSH2*, the corresponding cancer pair also failed to express *MSH2*, but expressed *MSH6* (Table 4). In five patients, a single cancer demonstrated MSI-H and the other showed MSI-L/MSS; one lost *MLH1* expression in both cancers, one showed loss of *MLH1* expression in the MSI-H cancers but not in the MSI-L/MSS cancer, while three patients expressed *MLH1*, *MSH2* and *MSH6* in both cancers. In seven patients, both cancers demonstrated MSI-L/MSS, a single cancer did not express *MSH2*, the remaining 13 cancers expressed *MLH1*, *MSH2* and *MSH6*.

In six patients, there was loss of expression of the same MMR gene in paired cancers. Their ages at diagnosis (median 54.5, range 40–73 years) were lower than in those with a single MSI-H cancer (median 75.5, range 60–84 years) (*P*=0.003).

### Methylation

Methylation analysis was carried out on all cancers that failed to express the *MLH1* gene protein. Of the single cancer group, three of the six (50%) MSI-H cancers and 11 of 17 (65%) MSI-H multiple cancers demonstrated promoter region methylation. Of three patients with paired cancers that failed to express *MLH1* in either cancer, two failed to demonstrate methylation (patients no. 4 and 7) and the other (patient no. 5) showed promoter region methylation in both cancers (Table 4).

### Mutational analysis

In 10 MSI-H multiple cancers that expressed the *MLH1* and *MSH2* protein, no mutations were identified in either gene.

## DISCUSSION

The incidence of MSI-H in multiple cancerss (52%) is significantly higher than in single cancers (13.5%), consistent with other reports (Sengupta *et al*, 1997; Brown *et al*, 1998; Masubuchi *et al*, 1999; Pedroni *et al*, 1999; Yamashita *et al*, 2000). The presence of patients with HNPCC, known to be associated with MSI-H in >90% of cancers, might account for this. Patients with multiple CRCs due to HNPCC are expected to demonstrate concordant MSI-H and loss of expression of the same mismatch repair gene in all cancers. We identified concordant MSI-H in only 25% of paired cancers, the remaining patients having either one or both MSI-L/MSS cancers. Since the incidence of HNPCC in patients with MSI-L/MSS cancers is very low (Terdiman *et al*, 2001), it is unlikely that HNPCC is responsible for the development of multiple cancers in 75% of patients. Of the 19 paired cancers from which tissue was available for immunohistochemical analysis, five patients demonstrated MSI-H with loss of a concordant MMR genes and one demonstrated concordant loss of *MLH1* expression in MSI-H and MSI-L cancers, suggesting that 31% of patients might have HNPCC. In addition, these patients were younger (median age 54.5 years) at the time of diagnosis than those with paired cancers in which only one demonstrated MSI-H (75.5 years). Since the median age of diagnosis of an HNPCC-related CRC is 42 years (Aarnio *et al*, 1995), this supports our hypothesis. However, when *MLH1* promoter region methylation analysis was performed on those MSI-H cancers that failed to express *MLH1*, one patient with a paired MSI-H cancer demonstrated concordant methylation. Since methylation accounts for loss of *MLH1* function in up to 90% of sporadic CRC and is uncommon in HNPCC (Wheeler *et al*, 2000), it is more likely that this patient has developed two sporadic cancers rather than having an underlying germline mutation, suggesting that, on combined immunohistochemistry and MSI analysis, only 26% of patients might have HNPCC. In addition, the relatively high incidence of methylation in *MLH1*-negative cancers (65%) throughout the group suggests that the majority of these patients have a sporadic MSI-H cancer and that an underlying germline mutation is not responsible.

Although the majority of the patients in our group do not appear to have classical HNPCC, the role of alternative MMR genes such as *MSH6*, PMS1, PMS2, MLH3 and EXO1 should be considered (Akiyama *et al*, 1997; Miyaki *et al*, 1997; Thompson *et al*, 2004). Since PMS1, MLH3 and EXO1 have only been implicated in the development of colorectal cancers in case reports (Liu *et al*, 1996;Hienonen *et al*, 2003;Thompson *et al*, 2004), they are unlikely to be implicated in the development of cancer in our group. However, *MSH6* mutations may be involved in 5% of all HNPCC families and give rise to atypical HNPCC with a delayed onset of cancer. Although extra-colonic cancers, particularly endometrial, appear to be more prevalent in this group (Plaschke *et al*, 2004), *MSH6* mutations have been implicated in the development of 22% of Amsterdam criteria-positive criteria patients with MSI-L cancers (Wu *et al*, 1999). Our finding that *MSH6* was expressed in all MSI-L and MSS cancers suggests that it is unlikely to play a significant role in this group. Of the MSI-H cancers, only five multiple cancers demonstrated loss of *MSH6* expression, four of these also failed to express *MSH2* or *MLH1*. In two cases, the paired cancer continued to express *MSH6* normally, while also failing to express *MSH2*, suggesting a secondary mutation possibly in the intragenic C_8_ mononucleotide repeat region of *MSH6* as a result of loss of *MLH1* or *MSH2* function. In only one case was *MSH6* expression lost in isolation, rendering a single patient a candidate for an underlying *MSH6* germline mutation. Mutational analysis in these cases may help clarify the role of *MSH6*. Mutations of PMS2 have been shown to be important in a small group of HNPCC patients (Thompson *et al*, 2004). Although primary mutations of PMS2 are uncommon, the inclusion of PMS2 immunohistochemistry may aid the identification of *MLH1* germline mutations in MSI-H cancers that continue to express *MLH1* (Thompson *et al*, 2004). The addition of PMS2 immunohistochemistry may have improved the low concordance between MSI-H and loss of MMR gene expression in multiple cancers (49%). The sensitivity of immunohistochemical analysis in our single cancers is in keeping with other series (Terdiman *et al*, 2001;Lindor *et al*, 2002), suggesting that this discrepancy is not due to experimental error. Missense mutations or in-frame deletions have been shown to account for the continued expression of a nonfunctioning MMR protein (Peltomaki and Vasen, 1997; Debniak *et al*, 2000; de Jong *et al*, 2004); therefore SSCP analysis of all *MLH1* and *MSH2* exons in 10 patients was undertaken to verify the immunohistochemistry result. Although intronic DNA was not analysed and SSCP is only sensitive for 70% of mutations, being particularly prone to miss large deletions (Grompe, 1993), we feel that it is unlikely that mutations, if present, would not have been identified in at least some of the 10 cancers studied. PMS2 analysis may elucidate the underlying genetic cause in this group.

In conclusion, although there is an increased incidence of MSI-H in those who develop multiple colorectal cancers, the majority are unlikely to be caused by HNPCC. In such cases, a combination of MSI analysis and immunohistochemistry analysis should be performed on both cancers to identify those who should undergo more extensive genetic evaluation.

## Figures and Tables

**Table 1:** Comparison between study and Thames Cancer Registry groups

	**Study group**	**Thames Cancer Registry**	**Statistical significance (*P*)**
Median age at diagnosis	72 (35–91) years	70 (32–94) years	0.11
*Site*			
Right colon	56	694	0.4
Left colon	51	986	
			
*Sex*			
Male	34	367	0.22
Female	44	387	
			
Metachronous	49	361	0.012
Synchronous	29	393	

**Table 2:**
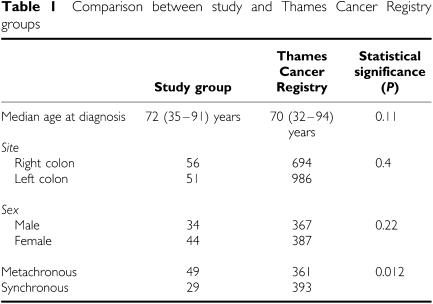
MSI status of paired cancers and statistical comparison of age at cancer presentation

**Table 3:** Immunohistochemistry results for multiple and single cancers

	**MSI-H**				**MSI-L/MSS**			
		**Negative staining**				**Negative staining**		
	**Positive staining**	***MLH1***	***MSH2***	***MSH6***	**Positive staining**	***MLH1***	***MSH2***	***MSH6***
*Multiple cancers*								
Number	22	14	7	5	28	3	1	0
Median age (range) (years)		72.5 (40–84) years				73 (35–91) years		
								
*Single cancers*								
Number	2	6	1	0	45	0	1	0
Median age (range) (years)		80 (68–89) years				73.5 (38–100) years		

**Table 4:** Immunohistochemistry and methylation data for ‘paired’ cancers

**Patient number**	**MSI status**	***MSH2* expression**	***MLH1* expression**	***MSH6* expression**	***MLH1* methylation**	**Age at presentation**
1	H	×	✓	×	—	55
	H	×	✓	✓	—	54
2	H	×	✓	×	—	40
	H	×	✓	✓	—	43
3	H	×	✓	✓	—	53
	H	×	✓	✓	—	42
4	H	✓	×	✓	×	50
	H	✓	×	✓	×	65
5	H	✓	×	✓	✓	67
	H	✓	×	✓	✓	73
6	H	✓	✓	✓	—	76
	H	✓	✓	✓	—	76
	H	✓	✓	✓	—	76
7	H	✓	×	✓	×	62
	L	✓	×	✓	×	62
8	H	✓	×	✓	✓	79
	H	✓	✓	✓	×	78
9	H	✓	×	✓	✓	82
	S	✓	✓	✓	×	73
10	H	✓	✓	✓	—	75
	L	✓	✓	✓	—	76
11	S	✓	✓	✓	—	84
	H	✓	✓	✓	—	84
12	H	✓	✓	✓	—	60
	L	✓	✓	✓	—	62
13	S	×	✓	✓	—	64
	L	✓	✓	✓	—	67
14	L	✓	✓	✓	—	73
	S	✓	✓	✓	—	73
15	L	✓	✓	✓	—	70
	L	✓	✓	✓	—	72
16	S	✓	✓	✓	—	77
	S	✓	✓	✓	—	78
17	L	✓	✓	✓	—	69
	L	✓	✓	✓	—	73
18	S	✓	✓	✓	—	57
	L	✓	✓	✓	—	59
19	L	✓	✓	✓	—	70
	L	✓	✓	✓	—	73

✓=positive, × =negative, —=not tested.
